# Lymphocyte and monocyte flow cytometry immunophenotyping as a diagnostic tool in uncharacteristic inflammatory disorders

**DOI:** 10.1186/1471-2334-10-205

**Published:** 2010-07-13

**Authors:** Helena Janols, Anders Bredberg, Irene Thuvesson, Sabina Janciauskiene, Olof Grip, Marlene Wullt

**Affiliations:** 1 The Department of Infectious Diseases, Skane University Hospital, Lund University, 20502 Malmo, Sweden; 2 The Cytometry Laboratory and The Department of Laboratory Medicine, Skane University Hospital, Lund University, 20502 Malmo, Sweden; 3 The Department of Pulmonology, Hannover Medical School, 30625 Hannover, Germany; 4 The Division of Gastroenterology and Hepatology, Department of Clinical Sciences Malmo, Skane University Hospital, Lund University, 20502 Malmo, Sweden

## Abstract

**Background:**

Patients with uncharacteristic inflammatory symptoms such as long-standing fatigue or pain, or a prolonged fever, constitute a diagnostic and therapeutic challenge. The aim of the present study was to determine if an extended immunophenotyping of lymphocytes and monocytes including activation markers can define disease-specific patterns, and thus provide valuable diagnostic information for these patients.

**Methods:**

Whole blood from patients with gram-negative bacteraemia, neuroborreliosis, tuberculosis, acute mononucleosis, influenza or a mixed connective tissue disorders, as diagnosed by routine culture and serology techniques was analysed for lymphocyte and monocyte cell surface markers using a no-wash, no-lyse protocol for multi-colour flow cytometry method. The immunophenotyping included the activation markers HLA-DR and CD40. Plasma levels of soluble TNF alpha receptors were analysed by ELISA.

**Results:**

An informative pattern was obtained by combining two of the analysed parameters: (i), the fractions of HLA-DR-expressing CD4+ T cells and CD8+ T cells, respectively, and (ii), the level of CD40 on CD14+ CD16- monocytes. Patients infected with gram-negative bacteria or EBV showed a marked increase in monocyte CD40, while this effect was less pronounced for tuberculosis, borrelia and influenza. The bacterial agents could be distinguished from the viral agents by the T cell result; CD4+ T cells reacting in bacterial infection, and the CD8+ T cells dominating for the viruses. Patients with mixed connective tissue disorders also showed increased activation, but with similar engagement of CD4+ and CD8+ T cells. Analysis of soluble TNF alpha receptors was less informative due to a large inter-individual variation.

**Conclusion:**

Immunophenotyping including the combination of the fractions of HLA-DR expressing T cell subpopulations with the level of CD40 on monocytes produces an informative pattern, differentiating between infections of bacterial and viral origin. Furthermore, a quantitative analysis of these parameters revealed the novel finding of characteristic patterns indicating a subacute bacterial infection, such as borreliosis or tuberculosis, or a mixed connective tissue disorder. The employed flow cytometric method is suitable for clinical diagnostic laboratories, and may help in the assessment of patients with uncharacteristic inflammatory symptoms.

## Background

A considerable number of patients display uncharacteristic inflammatory symptoms, and constitute a diagnostic and therapeutic challenge. The clinical history may be dominated by long-standing fatigue or pain, or by a prolonged fever. A number of reports on patients with inconclusive presentation and microbiological test results have shown that many of them will eventually be assigned with a diagnosis of tuberculosis or cytomegalovirus infection [1-4]. These uncharacteristic cases may therefore represent an early stage, or show an atypical presentation, of a mixed connective tissue disorder or an infection.

Blood lymphocyte immunophenotyping by flow cytometry is a routine diagnostic procedure for assessment of lymphoproliferative diseases and HIV patient immunodeficiency. More recently it has become part also of the monitoring of patients taking immune-modifying drugs such as the rituximab (Mabthera) anti-CD20 monoclonal antibody. The aim of the present study is to determine if an extended immunophenotyping of lymphocytes and monocytes, including cellular activation markers, can define disease-specific patterns, and thus provide valuable diagnostic information for patients with uncharacteristic inflammatory symptoms.

The immune response during experimental infection with a number of microbial agents has been investigated in great detail. For some prototype bacterial and viral infections data has also been collected from patients. Gram-negative enterobacteriacae strongly stimulate neutrophil phagocytosis and cytokine production by monocytes, in addition to effects on B and CD4+ T lymphocytes [5]. Another strong immunostimulator is Epstein-Barr virus (EBV), with as much as 50% of all peripheral blood T cells being specific for this virus during the acute phase of the infection [6]. The response to EBV has been reported to be much dominated by an increase in number and activation of CD8+ T lymphocytes [7,8]. However, for many clinically significant microbial agents there is information only on a limited number of cellular immune parameters. For example, the relative frequencies of CD4+ T helper cells and CD8+ T cytotoxic cells is known to become altered by many microbes, for example CD4+ T lymphocytopenia has been documented in some cases of tuberculosis [9].

## Methods

### Patients

The samples from patients with an infectious disease diagnosis were obtained at their first consultation. Their history was then less than two weeks for the cases with gram-negative septicemia/pyelonephritis (Gr-) (n = 10, 7 females, 3 males), EBV (n = 4, all females) or influenza (Inf) (n = 5, all males), but longer for some of the patients with tuberculosis (Tb) (n = 8, 4 females, 4 males) or neuroborreliosis (Bo) (n = 7, 4 females, 3 males). The disease duration of the mixed connective tissue disorder (MCT) (n = 5, 4 females, 1 male) cases was not determined. The final diagnosis was based on a combination of clinical symptoms and conventional testing using Swedish national QC approved culture, PCR and serology.

### Study design

Ethical permit was obtained from the local ethical committee at Lund University (Dnr 288/2007) and an informed consent was given by the participating patients.

### Blood analysis

Leukocyte concentration was determined using an LH750 machine (BeckmanCoulter, Hialeah, FL, USA), and plasma levels of C-reactive protein (CRP) with an automated immunoturbidimetric assay system (IMMAGE immunochemistry system, Beckman Coulter, Bromma, Sweden) with a minimum detectable dose of 0.2 mg/L, both using Swedish national QC approved clinical diagnostic methodology. Soluble TNF alpha receptors (sTNFR) were assessed by a quantitative ELISA according to the instructions provided by the manufacturer (R&D Systems, Minneapolis, MN USA). The mean minimum detectable dose were for sTNFRI 0.77 pg/ml and for sTNFRII 0.6 pg/ml, with an inter-assay variation of < 10%.

### Flow cytometry

Whole blood (50 μl) was incubated at room temperature with antibodies conjugated with the fluorochromes fluorescein isothiocyanate (FITC), phycoerythrin (PE), PE-Texas Red and PE-cyanine5 (PE-Cy5) to permit 4-colour analysis. The following four antibodies were added to each cell tube: CD8-FITC/CD4-PE/CD3-PE-Texas Red/HLA-DR-PE-Cy5, CD20-FITC/CD27-PE/CD19-PE-Texas Red/CD5-PE-Cy5, CD14-FITC/CD40-PE/CD3-PE-Texas Red/CD16-PE-Cy5, CD8-FITC/CD27-PE/CD45R0-PE-Texas Red/HLA-DR-PE-Cy5, CD2-FITC/CD14-FITC/CD15-FITC/CD20-FITC/CD11c-PE/CD45-PE-Texas Red/HLA-DR-PE-Cy5 (this tube was for determination of CD11c- HLA-DR+ plasmacytoid dendritic cells, and contained multiple FITC-labelled monoclonals in order to define lineage-negative cells), CD19-FITC/CD34-PE/CD45-PE-Texas Red/HLA-DR-PE-Cy5, respectively. Background autoflourescence was monitored with 4-colour isotypic non-specific mouse Ig, separately for lymphocytes and monocytes, and this PE-labelled mouse Ig was used also for setting the cut-off for HLA-DR-positive reaction. Antibody conjugates were from BeckmanCoulter and DAKO-Cytomation (Glostrup, Denmark). A no-wash no-lyse protocol was used. In-house solutions for lysis and fixation were used. At least 1,000 events were analysed in a FC500 BeckmanCoulter flow cytometer. Acquisition and analysis was made using the CXP software (BeckmanCoulter).

### Statistical analysis

The non-parametric Wilcoxon's two-sample rank test (the Mann-Whitney test) was used [10].

A *p*-value of < 0.05 was regarded as significant.

## Results

**Figure 1:**
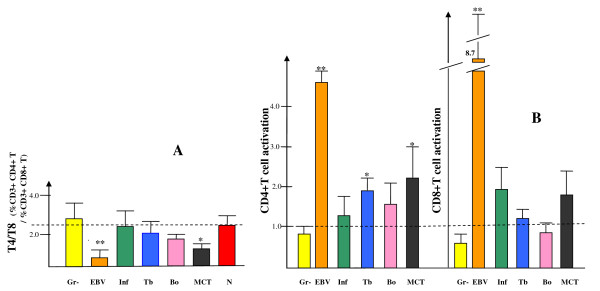
**T lymphocytes: (A), ratio of CD4+/CD8+ T cells; (B), activation within the CD4+ and CD8+ T cell subsets**. The result for T cell activation shows the percentage of HLA class II-expressing cells among all CD4+ T cells and CD8+ T cells, respectively. The indicated activation values are normalized with the result of the normal control individuals being expressed as 1.0, and the dashed lines indicate the level of the normal controls. Patient group codes: Gr-, septicaemia and/or pyelonephritis with gram-negative enterobacteria; EBV, Epstein-Barr virus; Inf, influenza; Tb, tuberculosis; Bo, neuroborreliosis; MCT, mixed connective tissue disorders; N, normal healthy controls. *p < 0.02 or 0.05, ** *p *< 0.01.

Several lymphocyte and monocyte subtypes were analysed with regard to their relative frequencies among peripheral blood leucocytes, and also for activation status, in order to evaluate if any of these data can be of clinical value. Patients with active and systemic EBV infection showed a clearly reduced CD4/CD8 ratio (0.3) as compared with the normal control group (N) (2.3) (Figure 1A). This effect was secondary to a strong increase in the number of CD8+ T cells, as indicated by the increased total blood lymphocyte count of 6.9 × 10 ^9 ^/ml (Table 1) (results from analysis of CD4+ T cell and CD8+ T cell percentages are not shown). The CD4/CD8 ratio of patients with the other infection types, including hospitalized cases of influenza, did not significantly differ from the controls (Figure 1A). The group of mixed connective tissue disorder patients (listed in figures as MCT) showed a low mean CD4/CD8 ratio (0.9).

**Table 1:** Leucocyte counts and plasma levels of C-reactive protein (CRP) and soluble TNF alpha receptors

Patient group	Neutrophils	Monocytes	Lymphocytes	CRP	sTNFRI	sTNFRII
**Gr-** (6.0-32.3)	**17.2** (0.6-1.3)	**0.8** (0.6-1.7)	**1.1** (114-332)	248** (5.5-10.3)	8.2** (6.9-21.3)	14.2**
**EBV** (1.4-6.0)	**4.6** (0.2-0.6)	**0.4** (3.2-18.5)	**6.9** (2-57)	30* (0.4-1.8)	**1.1** (8.4-13.0)	**10.7**
**Inf** (2.6-9.1)	**6.0** (0.1-0.9)	**0.5** (0.3-1.7)	**0.9** (55-215)	103** (1.0-2.0)	**1.5** (3.3-5.4)	**4.4**
**Tb** (3.4-9.1)	**5.8** (0.4-2.5)	**1.1** (0.9-4.3)	**2.1** (<2-150)	64* (0.8-11.3)	3.6* (2.3-20.8)	**7.7**
**Bo** (1.3-12.8)	**7.4** (0.4-0.8)	**0.6** (1.0-2.1)	**1.6** (<2-11)	**6** (0.8-2.3)	1.3* (2.6-3.4)	**3.0**
**MCT** (1.6-2.6)	**2.1** (0.1-0.6)	**0.4** (0.1-1.4)	**0.8** (2-74)	38*	**0.6**	**3.1**
**N** (0.08-1.3)	**1.7-8.0** (1.7-3.6)	**0.1-1.0**	**1.1-4.8**	**<2**	**0.4**	**2.5**

Leucocyte count (cells × 109/L), CRP (mg/L), TNF alpha receptors (pg/ml). For the MCT patient group TNF alpha receptors were determined for only one individual. Mean value and range are shown. For the N group (normal controls) leucocyte counts and CRP were not determined, and the displayed values represent the reference value ranges provided by the clinical laboratory performing the analysis of the patient groups. *P < 0.02, **P < 0.01.

**Figure 2:**
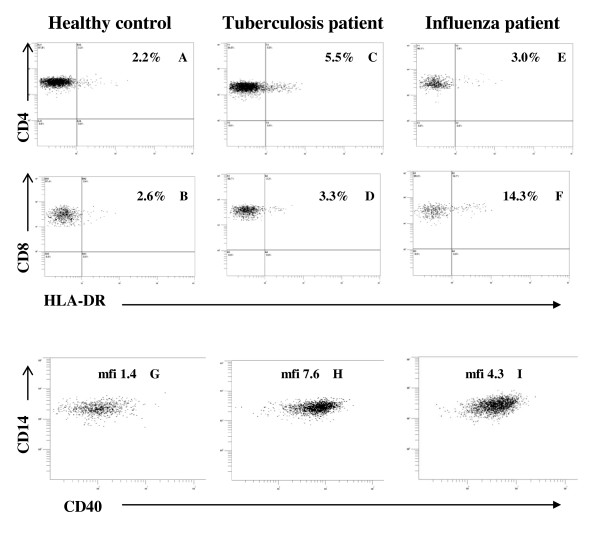
**Flow cytometry method for quantitation of T cell and monocyte activation status**. Representative dot plots from analysis of patient samples are shown. CD4+ T cells and CD8+ T cells gated on CD3+ CD4+ (A,C,E) and CD3+ CD8+ (B,D,F) lymphocytes from (A-B), a healthy control; (C-D), tuberculosis; (E-F), influenza; the inserted percentages indicate the fraction of HLA-DR+ cells. Monocyte plots gated on CD3- CD14+ monocytes from (G), a healthy control; (H), tuberculosis; (I), influenza; mfi = mean fluorescence intensity.

T cell activation, as determined by the presence of surface expression of HLA class II (known from *in vitro *to become detectable at 2-3 days after stimulation), was found to be strongly enhanced in the EBV patients, and notably not only in the CD8+ T cells but also markedly, although to a lesser degree, in the CD4+ T helper cells (Figure 1B). In the Gr- patient group (gram-negative bacteraemia or pyelonephritis) both of the T cell subtypes showed a low activation. In the second group of virus infection, influenza (Inf), activation was increased in the CD8+ T cells, and to some low extent also in the CD4+ T. However, for the Gr- and influenza groups, the differences as compared with the normal controls did not reach statistical significance. Conversely, both of the less acute bacterial infection patient groups showed normal CD8+ T activation, but an increased level in the CD4+ T cells, with significance for the Tb (tuberculosis) group at *p *< 0.02. There was no statistical significance for any of the differences observed between the Tb, Bo and MCT groups. Flow cytometry dot-plot raw data from representative cases are presented in Figure 2A-F, illustrating the strategy for determination of positivity for HLA class II expression on T cells.

**Figure 3:**
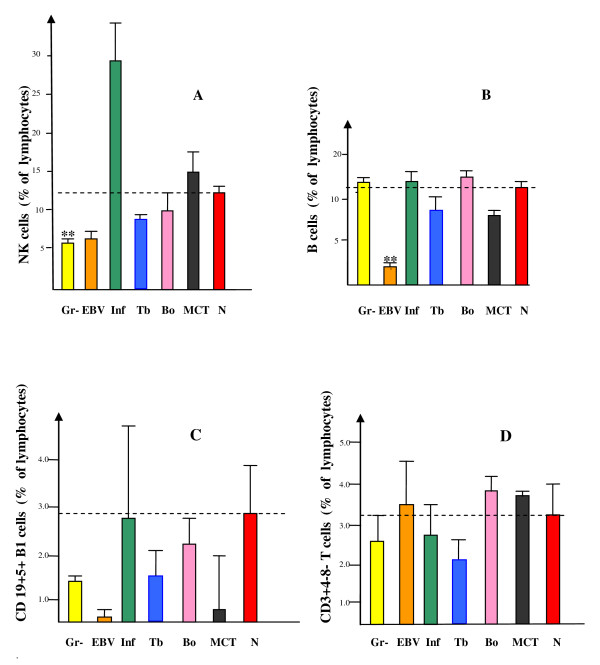
**NK cells, B cells and mucosal subtypes of B and T cells**. The results show the fraction among all blood lymphocytes of (A), NK cells; (B), B cells; (C), CD5+ B1 subtype of B cells; (D), gamma/delta TCR+ subtype of T cells as determined by a CD3+ CD4- CD8- phenotype. The dashed lines indicate the level of the normal controls. ** *p *< 0.01.

The fraction of NK cells was markedly elevated in three of the totally four analysed influenza patients yielding a mean % value more than double of that seen in any individual among the normal controls or the other patient groups; however, with no statistical significance due to a low value in the fourth patient presenting with a history of more than one week of subacute disease (Figure 3A) [11]. Interestingly, there was a statistically significant reduction of % NK cells in the Gr- group (*p *< 0.01) (Figure 3A). There were no significant differences in the fraction of B lymphocytes (Figure 3B). The cellular subtypes B1 among the B cells, and of gamma/delta T-cell receptor-positive T cells (herein defined by a CD3+ CD4- CD8- phenotype, shown by us previously to contain mostly gamma/delta T-cell receptor-positive T cells, results not shown), both being associated with mucosal immunity, did not show any signs to be a marker for any of the studied patient groups (Figure 3C-D). The low relative frequencies of B cell types and NK cells for EBV patients may be attributed to the marked expansion of CD8+ T cells leading to lymphocytosis, as described above for Figure 1A.

**Figure 4:**
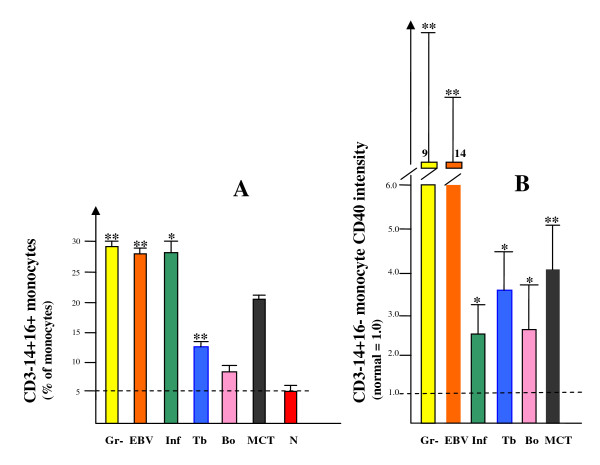
**Monocytes: (A), fraction among all monocytes of the CD16+ subtype; and (B), activation status as determined by CD40 expression**. The dashed lines indicate the level of the normal controls. *p < 0.02 or 0.05, ** *p *< 0.01.

Monocytes were investigated for several aspects; by a routine automated count of total monocytes (Table 1), by flow cytometry determination of the monocyte CD3- CD14+ CD16+ subtype (as % of all cells with light scatter properties typical for monocytes), and by CD40 intensity on the surface of the major CD3- CD14+ CD16- monocyte subtype, and finally by ELISA assay of plasma sTNFRI and sTNFRII [5,12,13]. Total monocyte count was elevated in most of the patients, as expected from their inflammatory clinical presentation; however, with a large inter-individual variation illustrated by the tuberculosis group with only two among the five studied individuals showing an increased total monocyte count (Table 1). Similarly, although plasma-CRP was elevated in most patients, there were cases presenting with values as low as 2.5 in the EBV group and < 2 mg/l among the tuberculosis and neuroborreliosis groups, providing no clear evidence of on-going infection (Table 1). In the control group (N) 5% of all monocytes were identified as belonging to the CD16+ subtype, whereas all the patient groups showed a higher frequency; for the Gr-, EBV and Inf groups at > 25% with statistical significance, and also among the Tb patients at the lower level of 13.7% but also with high significance (*p *< 0.01) (Figure 4A). Expression of CD40 (with CD40L = CD154 as its functional ligand on T cells) was evident on all CD14+ CD16- monocytes, but with a clearly increased density among all the studied patient groups (Figure 4B). The strongest expression was seen in the Gr- and EBV patients (*p *< 0.01), and a 2-3 fold elevation was evident in the Inf (*p *< 0.05), Tb (*p *< 0.02) and Bo (*p *< 0.05) groups. The MCT patients also showed an intermediate (4-fold) increase in CD40 expression. The CD16+ monocyte subset similarly showed an increase in CD40, but with a larger inter-individual variation (results not shown). Flow cytometry dot-plot raw data from representative cases are shown in Figure 2G-I, to illustrate the strategy for determination of the intensity of CD40 on the monocyte surface. Plasma levels of soluble TNF alpha receptors were elevated in most patients, but with a wide variation, and with clear statistical significance only for the Gr- patients (Table 1).

## Discussion

The present study was set up in order to determine whether multi-colour flow cytometry, now performed in many clinical diagnostic laboratories, can help us in the clinical assessment of patients with uncharacteristic inflammatory symptoms. Can it assist us by identifying lymphocyte and monocyte immunophenotypes suggesting an autoimmune disorder, or an on-going bacterial or viral infection?

An informative pattern was indeed obtained, by combining some of the analysed markers: the level of CD40 on monocytes with the fractions of HLA-DR-expressing CD4+ T cells and CD8+ T cells. For example, the obtained evidence suggests that an intermediate CD40 elevation in monocytes plus a large fraction of activated cells among the CD4+ T helper cells is indicative of subacute bacterial tuberculosis and borrelia infections. A similar pattern was seen among the mixed connective tissue disorder patients, however, with same levels of activation in the CD4+ T cells as in the CD8+ T cells. Statistical significance was reached for only some of the observed differences, and future studies on larger number of patients are needed in order to assess the full discriminatory power of these assays. Analysis of antinuclear antibodies and rheumatoid factor may therefore help to discriminate between infection and mixed connective tissue disorder in patients presenting with this type of pattern. Also NK cells provide valuable information since a marked increase was evident only in the influenza group (but not in the case of EBV, despite a clear virus pattern as determined by monocyte CD40 plus CD8+ T cells). We believe that activation markers (such as the informative CD40 and HLA-DR) for a principal reason are able to provide more reliable and easy-to-evaluate information than values expressed as percentage of all lymphocytes or leukocytes. This can be exemplified by the very strong increase in the number of CD8+ T cells in the EBV patients, causing the fractions of all other lymphocytes to be correspondingly decreased; thus, the low percentages of NK cells and of mucosal T and B cells cannot be assumed to reflect any influence by EBV on these cell types. In this context, it is interesting to turn the attention to the enterobacteriacae Gr- group: here the low values of NK cell fraction and T cell activation (both for CD4+ T and CD8+ T) cannot be considered to be secondary to lymphocytosis or any other of the assayed parameters. Instead, it suggests a direct effect on these cell types and, consequently, that less-needed immune functions can become down-regulated, in analogy with a study finding bacterial superinfections and a decreased granulocyte reactivity during a viral interferon-gamma response [14].

Our preliminary results from patients with uncharacteristic inflammatory disorders (n = 11, 8 females and 3 males), as defined by for example long-standing fatigue or pain, and no more specific diagnosis, show an elevated monocyte CD40 expression in most, but not all, cases (mean value 6 times higher than normal controls); an increased fraction of the CD14+ CD16+ monocyte subset (15% of all monocytes); and a most diverse T lymphocyte pattern. These patients have as yet not been followed-up with regard to additional tests or treatments.

Our study also shows that a number of potentially informative markers do not provide any discrimination. The fractions of B cells, CD5+ B cells and gamma/delta TCR+ CD3 + CD4- CD8- T cells (Figure 2B-D) did not show values different from the normal control persons; the latter two parameters are usually considered to play a role for mucosal immunity [15]. The CD14 and CD16 double-positive monocyte subpopulation showed much the same result as CD40 expression on classical CD14 + 16- monocytes, but with a large inter-individual variation limiting their value for clinical use. We assayed some additional cell types which did not provide any discrimination, without presenting the results; CD34 + CD45 ^low ^hematopoietic progenitor cells, present in peripheral blood at a fraction of 0.1% of all leukocytes and reported to become dramatically increased during an infection [16]; plasmacytoid dendritic cells [13], reported to increase during viral infections and in mixed connective tissue disorders; CD27 and CD45R0 memory and effector subtypes of CD8 + T cells, reported to be affected during persistent virus infections [17]. Likewise, CD69, CD71, as markers for T cell activation, as well as CD152 (CTLA-4) were found to be expressed only at low levels, and with no difference between patients and controls. No additional monocyte markers were investigated. It is known that activation antigen expression by T cells in vivo is determined by many factors such as body site, length of time of stimulation, and type of antigen [18]. We considered HLA-DR to be a suitable activation marker for the present study, not only because of its fluorescence intensity, but also with regard to its *in vivo *expression (whereas e.g. CD25, i.e. the IL2R, is more easily detected *in vitro*), and to its kinetics, peaking relatively late (whereas e.g. CD69 appears already 2 h after stimulation, to disappear at day 5).

We also assayed CD4 + T regulatory cells, but were not able to obtain high enough mean fluorescent intensities for CD25 and FoxP3 for a meaningful evaluation. Interestingly, we could not document any increase in CD154 (the CD40 ligand) on T cells, and therefore decided not to include CD154 in our standard panel. This finding may seem unexpected, since this surface marker on CD4 + T cells is known to interact with CD40 on antigen-presenting cells, found by us to become strongly up-regulated on monocytes [19]. Nevertheless, it is well-known that functional ligation by macrophages/monocytes with T cell CD40 ligand is important for their CD40 expression and effector functions such as TNF alpha and nitric oxide production [20]. Moreover, genetic studies have recently documented an important association between rheumatoid arthritis and CD40 variants, supporting the relevance of CD40 for inflammatory disease development [21]. Analysis of sTNFRs was also less informative due to a large variation, which may indicate that cellular activation markers in general have higher discrimination power than soluble cytokine and chemokine analytes, however, this remains for future studies to determine.

We have some evidence that the immunophenotype patterns found by us really provide superior information as compared with commonly used and more simple-to-assay parameters. In clinical practice a CRP value of 100 mg/l is often used as a crude cut-off, with a higher figure suggesting a bacterial rather than a viral origin of an infection. However, plasma-CRP was > 100 mg/L in some of the influenza patients, and < 100 mg/l for 14 of the 15 subacute bacterial tuberculosis and borreliosis patients, thus showing no reliable discrimination between viruses and bacteria. In addition, the range of CRP was wide within several patient groups, including some EBV, tuberculosis and neuroborreliosis patients presenting with a CRP of < 2 mg/l or 2 mg/l and thus giving no obvious indication of on-going infection. Other serum markers reported to be of use for rapid diagnosis of an infection were not assayed, such as procalcitonin in severe bacterial infections, neopterin and levels of cytokines or soluble cytokine receptors. The data obtained in the present study cannot exclude the possibility that the phase of the disease, i.e. acute versus chronic, rather than the type of disease, is the main determinant of the observed patterns. Future studies on a larger patient material may be helpful to clarify this issue. Interestingly, there was a normal monocyte count in all the EBV patients indicating that there is no simple relation between immune parameters assessed in peripheral blood and disease stage. However, there was a more than 3-fold increase in monocyte activation in all of the EBV patients, as compared with the highest CD40 level encountered among all of the control individuals. Likewise, although the tuberculosis group had an elevated mean number of monocytes, there was a large variation with only 2 of these 8 patients falling outside the normal range of monocyte count; in contrast the monocyte activation was increased in each patient.

## Conclusions

Immunophenotyping of lymphocytes and monocytes including quantities of the fractions of HLA-DR expressing T cells and the level of CD40 expression on monocytes can differentiate between acute bacterial and viral infections. Novel findings are presented, suggesting that it also provides some indication of a subacute bacterial infection, such as neuroborreliosis or tuberculosis; as well as of a mixed connective tissue disorder, respectively. The employed flow cytometric method is suitable for clinical diagnostic laboratories, and promises to be a source of valuable clinical information for patients with uncharacteristic inflammatory symptoms, guiding further diagnostic testing and providing a rationale for ex juvantibus therapy. Further studies involving a larger number of patients are warranted, in order to determine the full clinical discriminatory power of this diagnostic tool.

## Competing interests

The authors declare that they have no competing interests.

## Authors' contributions

MW initiated the study. AB, HJ, MW, OG and SJ designed the experimental protocol. AB, HJ and IT analysed the experimental data. AB and HJ wrote the manuscript. All authors read and approved the final manuscript.

## Pre-publication history

The pre-publication history for this paper can be accessed here:

http://www.biomedcentral.com/1471-2334/10/205/prepub

## References

[B1] Persson L Dahl H Linde A Engervall P Vikerfors T Tidefelt U Human cytomegalovirus, human herpesvirus-6 and human herpesvirus-7 in neutropenic patients with fever of unknown origin Clin Microbiol Infect 2003 9 640 644 10.1046/j.1469-0691.2003.00578.x 12925104

[B2] Ergonul O Willke A Azap A Tekeli E Revised definition of 'fever of unknown origin': limitations and opportunities J Inf 2004 50 1 5 10.1016/j.jinf.2004.06.007 15603833

[B3] Chin C Chen YS Lee SJ Wann SR Lin HH Huang CK Tsai HC Kao CH Yen MY Liu YC Fever of unknown origin in Taiwan Infection 2006 34 75 80 10.1007/s15010-006-5010-2 16703296

[B4] Manfredi R Calza L Chiodo F Primary cytomegalovirus infection in otherwise healthy adults with fever of unknown origin: a 3-year prospective survey Infection 2006 34 87 90 10.1007/s15010-006-5012-0 16703298

[B5] Abbas AK Lichtman AH Cellular and Molecular Immunology 2003 5 Philadelphia, PA: W.B. Saunders

[B6] Thorley-Lawson DA Epstein-Barr virus: exploiting the immune system Nature Rev Immunol 2001 1 75 82 10.1038/35095584 11905817

[B7] Roos MTL van Lier RAW Hamann D Knol GJ Verhoofstad I van Baarle D Miedema F Schellekans PTA Changes in the composition of circulating CD8+ T cells during acute Epstein-Barr and immunodeficiency virus infections in humans J Inf Dis 2000 182 451 458 10.1086/315737 10915075

[B8] Lima M dos Angos Texeira M Queiros ML Santos AH Goncalves C Correia J Farinha F Mendonca F Soares JMN Almeida J Orfao A Justica B Immunophenotype and TCR-Vbeta repertoire of peripheral blood T-cells in acute infectious mononucleosis Blood Cells Mol Dis 2003 30 1 12 10.1016/S1079-9796(03)00014-7 12667982

[B9] Uppal SS Tewari SC Verma S Dhot PS Comparison ofCD4 and CD8 lymphocyte counts in HIV-negative pulmonary TB patients with those in normal blood donors and the effect of antitubercular treatment: hospital-based flow cytometric study Cytometry B Clin Cytom 2004 61 20 26 10.1002/cyto.b.20018 15351978

[B10] Altman DG Practical statistics for medical research 1990 Chapman and Hall. London

[B11] Strowig T Brilot F Munz C Noncytotoxic functions of NK cells: direct pathogen restriction and assistance to adaptive immunity J Immunol 2008 180 7785 7791 18523242 10.4049/jimmunol.180.12.7785 PMC2575662

[B12] Grip O Bredberg A Lindgren S Henriksson G Increased subpopulations of CD16(+) and CD56(+) blood monocytes in patients with active Crohn's disease Inflamm Bowel Dis 2007 13 566 72 10.1002/ibd.20025 17260384

[B13] Auffray C Sieweke MH Geissman F Blood monocytes: development, heterogeneity, and relationship with dendritic cells Ann Rev Immunol 2009 27 669 692 10.1146/annurev.immunol.021908.132557 19132917

[B14] Navarini AA Lang KS Verschoor A Recher M Zinkernagel AS Nizet V Odermatt B Hengartner H Zinkernagel RM Innate immune-induced depletion of bone marrow neutrophils aggravates bacterial infections Proc Natl Acad Sci (USA) 2009 106 7107 12 10.1073/pnas.0901162106 PMC2678456 19351895

[B15] Ruiz P Geraldina N Peripheral gamma delta T-cell populations in HIV-infected individuals with mycobacterial infection Cytometry 1995 22 211 216 10.1002/cyto.990220308 8556952

[B16] Massberg S Schaerli P Knezevic-Maramica I Kollnberger M Tubo N Moseman EA Huff IV Junt T Wagers AJ Mazo IB von Andrian UH Immunosurveillance by hematopoietic progenitor cells trafficking through blood, lymph and peripheral tissues Cell 2007 131 994 1008 10.1016/j.cell.2007.09.047 18045540 PMC2330270

[B17] Appay V Dunbar PR Callan M Klenerman P Gillespie GMA Papagno L Ogg GS King A Lechner F Spina CA Little S Havlir DV Richman DD Gruener N Pape G Waters A Easterbrook P Salio M Cerundolo V McMichael AJ Rowland-Jones SL Memory CD8+ T cells vary in differentiation phenotype in different persistent virus infections Nat Med 2002 8 379 385 10.1038/nm0402-379 11927944

[B18] Amlot PL Tahami F Chinn D Rawlings E Analysis by two-colour flow cytometry of umbilical cord blood, Activation antigen expression on human T cells. I. adult blood and lymphoid tissue Clin Exp Imunol 1996 105 176 182 10.1046/j.1365-2249.1996.d01-722.x PMC2200463 8697627

[B19] Elgueta R Benson MJ de Vries VC Wasiuk A Guo Y Noelle RJ Molecular mechanism and function of CD40/CD154 engagement in the immune system Immunol Rev 2009 229 152 172 10.1111/j.1600-065X.2009.00782.x 19426221 PMC3826168

[B20] Suttles J Stout RD Macrophage CD40 signaling: a pivotal regulator of disease protection and pathogenesis Semin Immunol 2009 21 257 264 10.1016/j.smim.2009.05.011 19540774

[B21] Criswell LA Gene discovery in rheumatoid arthritis highlights the CD40⁄ NF-jB signaling pathway in disease pathogenesis Immunol Rev 2010 233 55 61 10.1111/j.0105-2896.2009.00862.x 20192992

